# Fibrosarcoma of the ethmoid sinus: A rare entity

**DOI:** 10.1016/j.ijscr.2019.05.023

**Published:** 2019-05-14

**Authors:** Najib Zouhair, Anass Chaouki, Amine Ballage, Redalah Elarabi Abada, Sami Rouadi, Mohamed Roubal, Mohamed Mahtar

**Affiliations:** Department of Otorhinolaryngology, Hospital of 20 August, University of Hassan II, Casablanca, Morocco

**Keywords:** Fibrosarcoma, Ethmoid, Endoscopy, Navigation, Histopathology

## Abstract

**Introduction:**

Ethmoidal fibrosarcoma is an extremely rare tumor which represent less than one percent of all malignancies in this anatomical area, the case of a 13-year-old is reported here.

**Presentation of case:**

A 13-year-old boy, his medical history was marked exclusively before presented to our hospital by an exophthalmia. Rhinoscopic examination showed well-defined pink mass at the level of ethmoid. Computed tomography was performed to showed local and regional extension. Treatment was an endoscopic excision of the tumor using a navigation system. The final diagnosis was made on pathologic examination. Radiotherapy was recommended by multidisciplinary staff but the patient refused.

**Discussion:**

These tumors occur in extremities most frequently and Ethmoidal localization is extremely rare and have no specifics symptoms, so the rhinoscopic examination is necessary for any chronic nasal symptom. Its treatment is not codified because of its rarity and its prognosis is unknown.

**Conclusion:**

It’s important knowledge about this pathology because of its rarity and its therapeutic and prognostic difficulties.

## Highlights

• Ethmoidal fibrosarcoma is an extremely rare tumor.
• Mostly misdiagnosed because of none specifics symptoms, in this case patient didn’t complaint any nasal symptom.
• Treatment is not codified and prognosis is unknown.

## 1 Introduction

Fibrosarcoma is a malignant neoplasm of the fibroblastic origin, most commonly occurring from the soft tissue of extremities. Occurrences in the head and neck are not common, accounting for <1% of the malignancies in this anatomical area [1]. Ethmoid sinus location is an extremely rare event [2]. Clinical presentation is not specific. The case of a fibrosarcoma of ethmoid sinus in a 13-year-old boy presenting with exophthalmia is reported here.

## 2 Case report

A 13-year-old boy presented with a one year history of exophthalmia in the left eye, without any nasal symptoms. There was no history of local trauma or systemic disease, and he was operated on 6 months ago under a rhinoscopic approach when a marsupialization of the cyst was done with pathological examination showing characteristics of an aneurysmal cyst. He was actually admitted for recidivism of the same lesion.

**Fig. 1:**
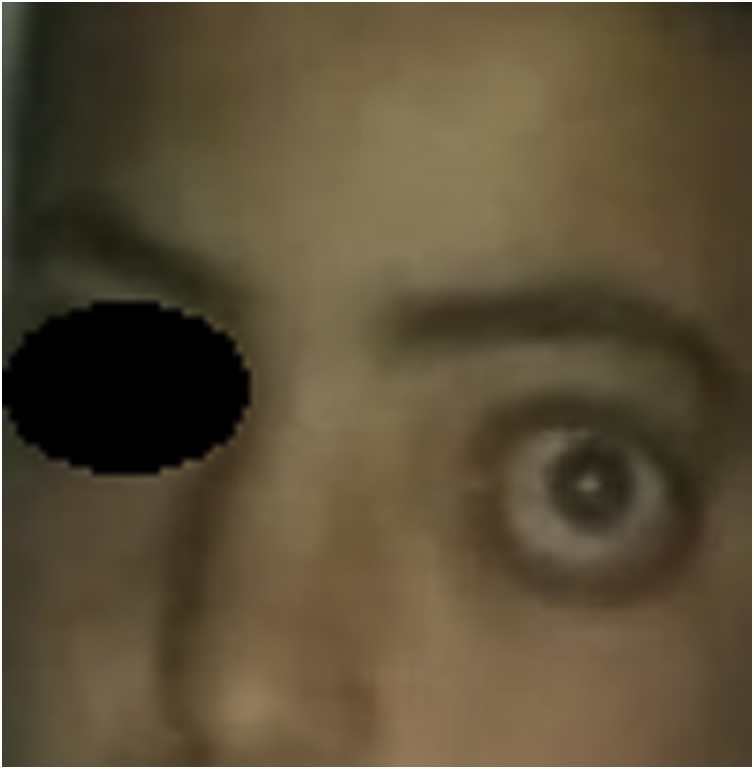
Exophthalmia of the right eye.

Clinical examination showed exophthalmia of the left eye without loss of visual acuity (Fig. 1).

**Fig. 2:**
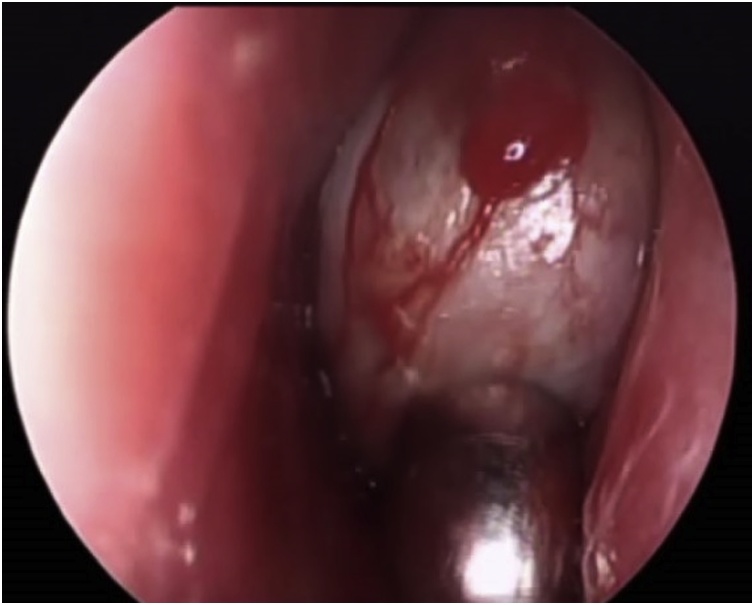
Rhinoscopy showing a well-defined mass smooth and pink.

Rhinoscopy found a well-defined mass, sitting at the level of the left ethmoidal sinus, smooth and pink. The other side was normal (Fig. 2).

**Fig. 3:**
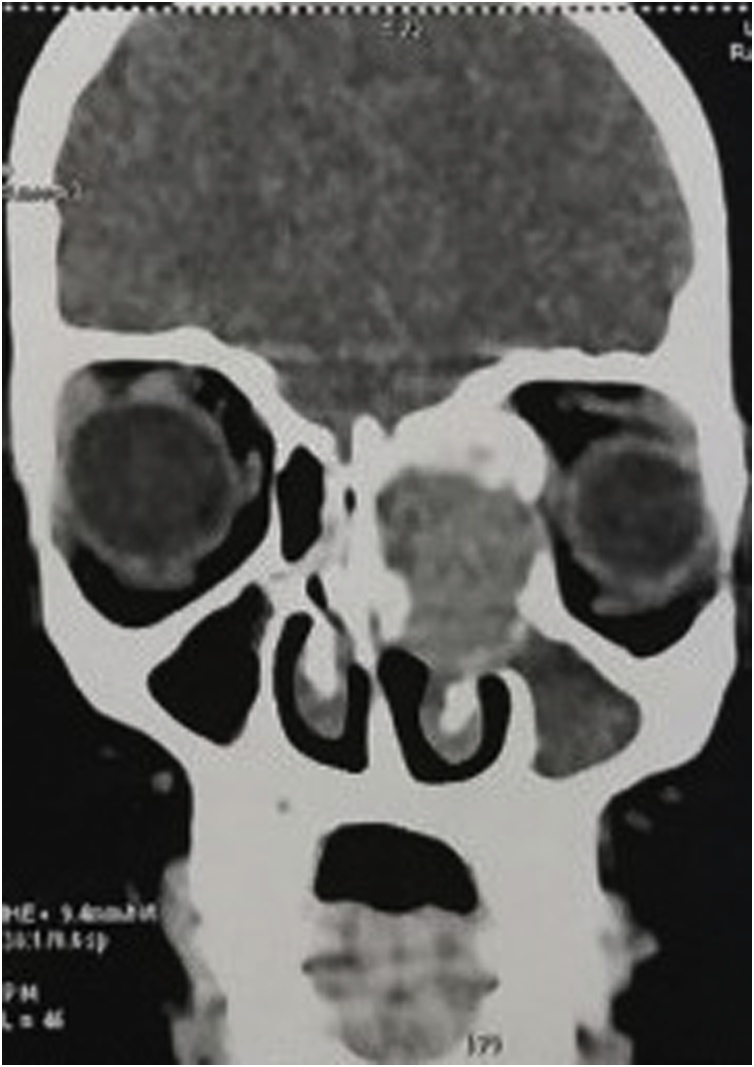
Oval lesion replacing the ethmoidal cells measuring 39 × 23 × 35 mm.

Tomodensitometry showed an oval multiloculated lesion more extensive in the anteroposterior plan replacing the ethmoidal cells measuring 39 × 23 × 35 mm. This lesion has regular walls, duplicated by place. Its content is made of multiple stalls with a liquid level realized by blood outside as it pushes the globe and the right internal muscles without signs of invasion, responsible for an exophthalmia grade I. Inside it fills the nasal fossa and pushes the septum without a free interface. At the top it displaces the ethmoidal roof inward without endocranial invasion. It is responsible for fluid retention at the level of the left maxillary sinus, and through posterior ethmoid cells invasion it is responsible for the narrowing of the optical channel, whereas the frontal and sphenoidal sinuses are free (Fig. 3).

**Fig. 4:**
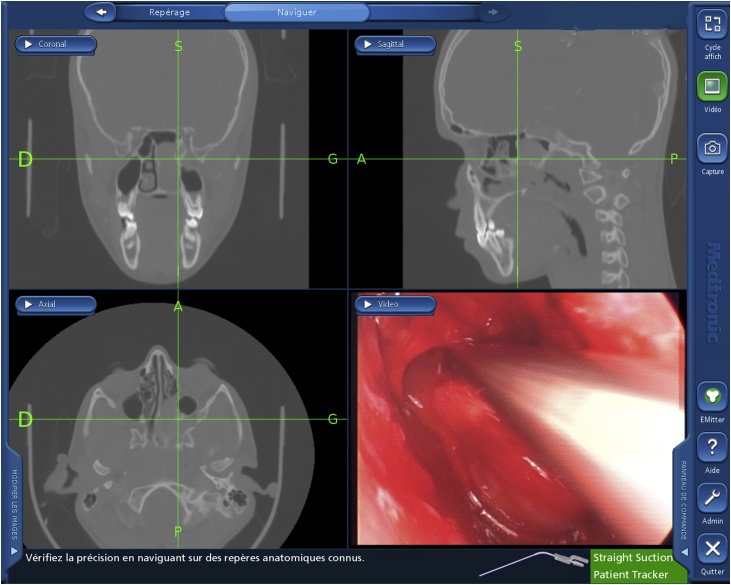
Navigation showing internal orbital wall in the three plan of space.

Surgical intervention involved the total excision of the tumor with all its walls, in addition to the orbital medial wall and its periorbital by endoscopic approach. This was assisted by ENT navigation system which was helpful to determine the skull base, the orbit and the carotid canal because landmarks are modified by the tumor (Fig. 4).

**Fig. 5:**
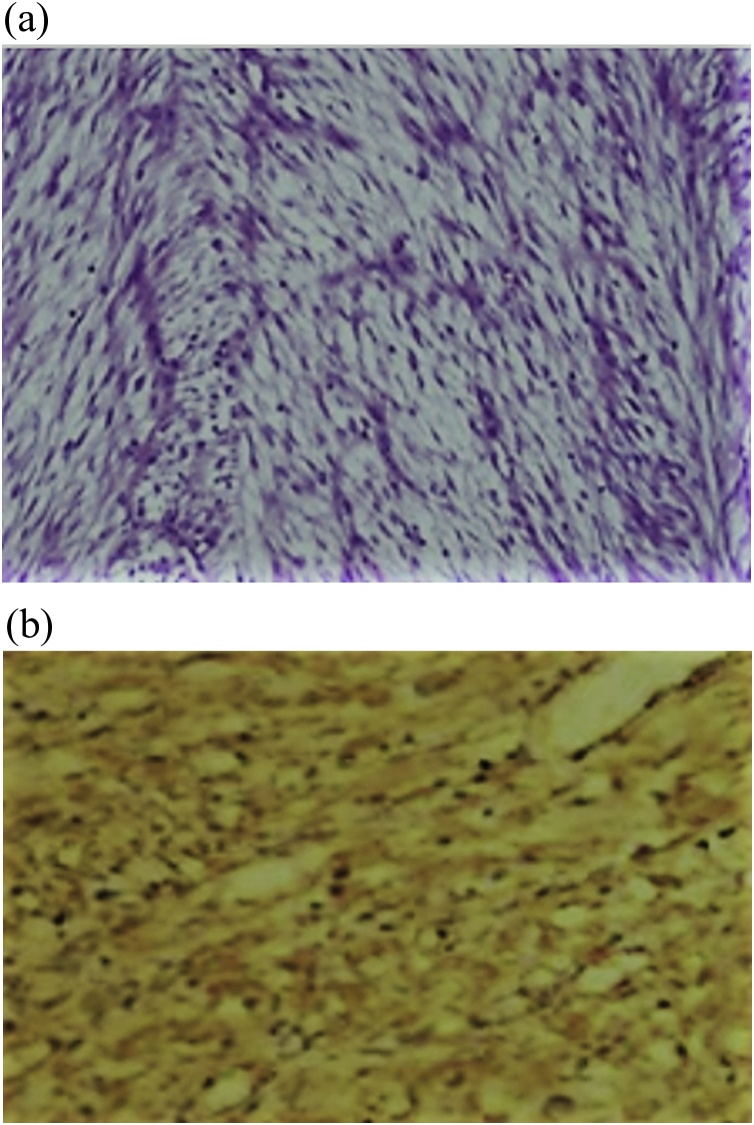
(a): proliferation in herring bone with multinucleate elements, (b): The tumor cells were positive for vimentin.

Histopathology found fusocelular carcinomatous proliferation in the herring bone with calcification, presence of multinucleate elements showing atypia estimated at 3/10 fields. Those tissues are negative for desmin and CD 34 and positive for vimentin (Fig. 5).

The multidisciplinary team deciding on the treatment options included surgeons, oncologists, radiotherapists and radiologists. The decision was made to treat the patient with radiotherapy, but the patient refused. The alternative was to closely monitor the patient, with monthly clinical and endoscopic examinations. No recidivism was actually found after one year of follow-up.

## 3 Discussion

Fibrosarcomas may develop in any mesenchymal tissue in which fibroblasts are present; they are most frequently located in the extremities and the trunk. Fibrosarcoma can arise in soft tissues or within bone. In the head and neck region, soft tissue sarcomas are extremely rare, accounting for less than 1% of all neoplasms [3].

It is more frequent in the fifth and sixth decades of age but cases in children and adolescents were also described in the literature [4,5].

Infantile fibrosarcoma includes less than one percent of childhood tumors and about 10 percent of soft tissues sarcomas [6,7].

Some authors have mentioned a cut‑off age of 2 years [8,9], whereas others have suggested a cut‑off age up to 5 years [10,11].

2 years was suggested to be the cut‑off age for infantile fibrosarcoma by the World Health Organization (WHO) [12].

Early signs and symptoms are unfortunately vague and may mimic minor upper respiratory infections. The most common presentation for sinonasal fibrosarcomas includes nasal airway obstruction, pain, epistaxis and hypoesthesia, not one of these symptoms was present in our patient [13].

Pathologic examination is the only way of diagnosis after imaging studies. Histologically, sinonasal fibrosarcomas are composed of hypercellular tumors with spindle shaped thin cells arranged in typical herring bone pattern [14]. When this histologic picture is diffuse and uniform, diagnosis can be made even without ancillary studies [15]. These tumors are negative for pancytokeratin, desmin, S100 and SMA. They are only positive for vimentin [16].

Surgery remains the mainstay for the treatment of fibrosarcomas. Wide local excision with radical margins is generally recommended [17,18]. Adjuvant radiotherapy is also used in positive surgical margins or macroscopically incomplete excision [19]. In this case the patient refused radiotherapy so the multidisciplinary decision was to opt for a complete surgical excision with a monthly follow-up.

The greatest advantage of endoscopic surgery assisted by navigation for malignant tumors is the reduction in morbidity and length of stay, but surgical margins are not always controlled.

## 4 Conclusion

Fibrosarcoma is very rare tumor, especially head and neck localization, symptoms are not specific, so the rhinoscopic examination is necessary when investigating any chronic nasal symptom or exophthalmia. Its treatment and prognosis is not codified because of its rarity.

## Conflicts of interest

Authors do not declare any conflict of interest.

## Sources of funding

This research did not receive any specific grant from funding agencies in the public, commercial, or not-for-profit sectors.

## Ethical approval

This study is exempt from ethnical approval in our institution.

## Consent

Written informed consent was obtained from the patient's parents for publication of this case report and accompanying images. A copy of the written consent is available for review by the Editor-in-Chief of this journal on request

## Author contribution

Dr Zouhair and dr chaouki have participated in the papers writing and images treatement, Pr Rouadi, Pr Abada, Pr Roubal and Pr Mahtar in the rereading and final approval of the manuscript upon submission.

## Registration of research studies

None.

## Guarantor

Zouhair Najib.

## Provenance and peer review

Not commissioned, externally peer-reviewed.
